# BEST PRACTICES IN COMMUNICATING ABOUT PALLIATIVE CARE TO PATIENTS AND PHYSICIANS

**DOI:** 10.1093/geroni/igac059.848

**Published:** 2022-12-20

**Authors:** Alexis Coulourides Kogan, Anna Rahman, Sindy Lomeli, Susan Enguidanos

**Affiliations:** Keck School of Medicine of USC, Alhambra, California, United States; University of Southern California, Los Angeles, California, United States; University of Southern California, Los Angeles, California, United States; University of Southern California, Los Angeles, California, United States

## Abstract

Home-based palliative care (HBPC) programs are proliferating across the U.S, yet face significant, documented challenges in promoting uptake of services and sustaining sufficient patient referrals. There is a tremendous need to understand effective methods for engaging physicians, patients, and caregivers in palliative care. Thus, the purpose of this study was to elicit best practices in how to communicate about HBPC to both healthcare providers and patients/caregivers. Focus groups with nine California-based HBPC organizations were conducted between January and April 2020. Discussions lasted approximately 54 minutes, were guided by a semi-structured protocol, audio recorded, and transcribed verbatim. Thematic analysis was used to identify themes and codes from the data. Twenty-five interdisciplinary HBPC staff members participated in a focus group. Most identified as white (76%), female (76%), and working in their current position for five years or less (56%). Three themes were identified from the data: (1) value of relationships; (2) communication do’s and don’ts; and (3) need for education. Participants discussed actionable recommendations for each theme. Study findings highlight several best practices for HBPC programs to communicate- and foster relationships with healthcare professionals and patients/families about palliative care, with education at the crux. Lessons learned about key words and phrases to say and to avoid are particularly valuable for budding HBPC programs. Our results suggest that HBPC providers exert enormous efforts to increase patient referrals and enrollment through strategic, continuous outreach and education to physicians, patients, and their caregivers; however, palliative care educational interventions are needed.

